# Association between Smoking Habits and *Dopamine Receptor D2 Taq*I A *A2* Allele in Japanese Males: a Confirmatory Study

**DOI:** 10.2188/jea.12.297

**Published:** 2007-11-30

**Authors:** Nobuyuki Hamajima, Hidemi Ito, Keitaro Matsuo, Toshiko Saito, Kazuo Tajima, Masahiko Ando, Kimihide Yoshida, Takashi Takahashi

**Affiliations:** 1 Division of Epidemiology and Prevention, Aichi Cancer Center Research Institute.; 2 2nd Department of Internal Medicine, Nagoya City University School of Medicine.; 3 Nagoya University Graduate School of Medicine.; 4 Department of Preventive Medicine, Nagoya University Graduate School of Medicine.; 5 Department of Pulmonary Medicine, Aichi Cancer Center Hospital.; 6 Division of Molecular Oncology, Aichi Cancer Center Research Institute.

**Keywords:** smoking, dopamine receptor D2, polymorphism, PCR-CTPP

## Abstract

Our previous study showed that *A2* allele of dopamine *D2 (DRD2) Taq*I A polymorphism related to smoking habits, which was opposed to the results of studies for Caucasians. In order to confirm our finding, a similar study was conducted for the first-visit outpatients of Aichi Cancer Center Hospital, who participated in HERPACC-II (Hospital-based Epidemiologic Research Program at Aichi Cancer Center - II). Among consecutive 1,577 first-visit patients between November 2000 and February 2001, 800 patients provided a 7ml of peripheral blood. Smoking habit data were available for 798 participants. Excluding five participants aged < 20 years or ≥ 80 years, the remaining 793 participants (346 males and 447 females) were analyzed. The *DRD2* genotype was determined by a new method, polymerase chain reaction with confronting two-pair primers (PCR-CTPP). In males, current smokers were 35.3% of individuals with *A1A1* genotype, 43.1% of individuals with *A1A2* genotype, and 57.0% of individuals with *A2A2* genotype, while in females, they were 19.6%, 14.6%, and 10.9%, respectively. Age-adjusted odds ratio (OR) of current smoking relative to *A1A1* was 1.61 (95% confidence interval, 0.71-3.46) for *A1A2* and 2.32 (1.02-5.29) for *A2A2* in males, and 0.72 (0.32-1.61) and 0.51 (0.22-1.18) in females, respectively. The present study indicated that Japanese males with *A2A2* genotype have a higher risk of being current smokers. No association for Japanese females suggested that female smoking behavior is differently affected in biological and/or psychological manner.

The associations with genetic polymorphisms propose a new insight that smoking behavior may be constitutionally influenced through biological mechanisms.^1^ To date, polymorphisms pertaining to neurotransmitters,^2^^-^^9^ nicotine metabolism,^10^ and inflammation^11^ have been reported to have the associations with smoking habits, though inconsistent findings have also been observed.^12^^-^^14^ Since these associations with genetic polymorphisms have possible biological reasoning, the consistent findings in epidemiologic studies are important to confirm the causal link with smoking habits.

Concerning dopamine receptor *D2 (DRD2) Taq*I A biallelic polymorphism with *A1* and *A2* alleles, four studies have been conducted for the association with smoking habits among non-Hispanic Caucasians.^2^^-^^4^^,^^12^ Three of the four showed an association with *A1* allele (or closely linked *B1* allele of *Taq*I B polymorphism), while the other one reported an insignificant association with *A2* allele.^12^ We previously reported a significant association with *A2* allele of *DRD2 Taq*I A for Japanese.^15^ The finding suggests that there are different links to other *DRD2* polymorphisms or other genes responsible to smoking behavior. Another possible explanation is interactions with other genes or lifestyle/psychological factors, which convert the effect of the alleles. Although associations with the different allele could be observed among different ethnic groups, confirming the association for different Japanese subjects is the first step to discuss the above listed possibilities.

This study aimed to confirm our previous finding that the *A2* allele relates to smoking behavior for Japanese. The previous study was conducted for re-visit non-cancer outpatients of Aichi Cancer Center Hospital, and this time for first-visit outpatients of the same hospital. In this study, never smokers were defined as those who smoked less than 100 cigarettes in the past, current smokers as those who smoked in the past one year, and former smokers as those who quit smoking more than one year before the questionnaire study.

*DRD2* has been reported to have 20 polymorphisms; *Taq*I A, *Taq*I B, *Taq*I D, *Eco*RI, *Bcl*I, *Mbo*I, and *Hinc*II restriction fragment length polymorphisms (RFLP), *GA* and *GT* tandem repeat polymorphisms, a C-to-G polymorphism not genotyped by PCR-RFLP listed in Genbank (Accession No. AF050737), as well as a functional polymorphism -141C Ins/Del in the promoter region,^16^ three missense polymorphisms (Val96Ala, Pro310Ser, and Ser311Cys),^17^ and six silent variants (44Leu,^17^ 141Leu,^18^ 255Val,^19^ 319Pro,^20^ 313His,^18^ and 367Lys^19^). The *Taq*I A and *Taq*I B are linked closely,^4^^,^^15^ and we found that -141C Ins/Del polymorphism was not associated with smoking habits.^15^ In this paper, *Mbo*I polymorphism located in intron 2 was examined for a subset of the subjects on an exploratory purpose, as well as *Taq*I A in the 3′ untranslated sequence of exon 8.

## MATERIALS AND METHODS

### Study subjects

Subjects were first-visit patients of Aichi Cancer Center Hospital who were consecutively invited to lifestyle questionnaire and peripheral blood donation in the framework of HER-PACC-II.^21^ The participants in HERPACC-II during November 2000 and February 2001 were sampled. Among 1,577 first-visit outpatients, 800 provided a 7ml of peripheral blood. Data on smoking habits were available for all but two. Four participants aged less than 20 and one aged 80 years or over were excluded from the analysis. The remaining were 793 participants (346 males with mean age 55.8 years and standard deviation 12.1 years, and 447 females with mean age 50.2 years and standard deviation 13.1 years). The first 395 outpatients (174 males and 221 females) were used for exploratory analysis of *Mbo*I polymorphism. In this hospital, cancer patients are about 20% of the first-visit outpatients. Since this study examined the association with smoking habits defined at the time one year before their visit, cancer patients were included in the study subjects.

This study had been approved by the Ethical Committee at Aichi Cancer Center before the study started (Ethical Committee Approval Numbers 41-2).

### Genotyping

DNA was extracted from 200 *μ*l buffy coat preserved at -80°C by QIAamp DNA Blood Mini Kit (QIAGEN Inc., Valencia, CA). The genotyping was conducted by a novel PCR technique, PCR-CTPP (polymerase chain reaction with confronting two-pair primers).^22^^,^^23^ The primers were F1: 5′ TGA GCC ACC ACG GCT GG, R1: 5′ CAT CCT CAA AGT GCT GGT CG, F2: 5′ AGC TGG GCG CCT GCC TT, and R2: 5′ CTC TTG GAG CTG TGA ACT GG for *Taq*I A polymorphism, and F1: 5′ GAG AAA TGA TGC TTT CGG AAA AAT, R1: 5′ CAT GTG TCA GGC ACT GT, F2: 5′ GAT ATA AGC ATC AAG TGT TTG GAT, and R2: 5′ GGC ATC CAG GCA TCA TT for *Mbo*I polymorphism. The underlined are the sites of single nucleotide polymorphisms.

Genomic DNA (30ng to 100ng) was used in a volume of 25 *μ*l with 0.1 mM dNTPs, 12.5 pmol of each primer, 0.5 units of “AmpliTaq Gold” (Perkin-Elmer Corp., Foster City, CA), and 2.5 *μ*l 10× PCR Buffer including 15mM MgCl_2_. A 2.5 *μ*l of glycerol was added in genotyping for *Taq*I A polymorphism, not for *Mbo*I polymorphism. PCR for *Taq*I A was conducted as follows; a 10 min of initial denature at 95°C, 30 cycles of 1 min at 95°C, 1 min at 56°C, and 1 min 72°C, and a 5 min of final extension at 72°C. The condition for *Mbo*I was same but annealing temperature at 60°C.

**Figure 1.**
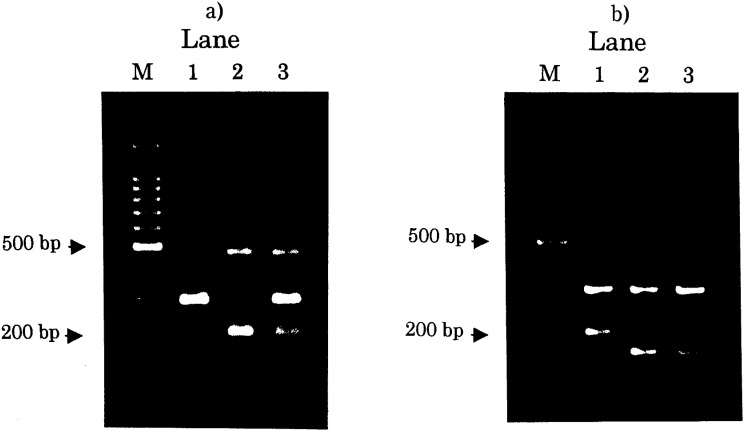
Representative examples of agarose gel electrophoresis; a) *DRD2 Taq*I A polymorphism, lane M for a 100-bp ladder marker, lane 1 for *A1A1* genotype, lane 2 for *A2A2* genotype, and lane 3 for *A1A2* genotype; b) *DRD2* *Mbo*I polymorphism, lane M for a 100-bp ladder marker, lane 1 for *AA* genotype, lane 2 for *TT* genotype, and lane 3 for *AT* genotype. A 493-bp common band is not amplified for lane 1 of *DRD2 Taq*I A. This phenomenon is often observed and does not disturb correct genotyping.

All PCR products were visualized on a 2% agarose gel with ethidium bromide staining. Genotyping of *Taq*I A is 292 bp for *A1* (*T*) allele and 207 bp for *A2* (*C*) allele with a 493-bp common band, and that of *Mbo*I 196 bp for *A* allele and 154 bp for *T* allele with a 310-bp common band, as demonstrated in Figure 1.

### Statistical analysis

Odds ratios (ORs) and 95% confidence intervals (CIs) were estimated by an unconditional logistic model with computer program STATA Version 7 (STATA Corporation, College Station, TX). Age-adjustment for the ORs was conducted as a continuous variable. The fitness for Hardy-Weinberg equilibrium was examined by “genhwi” command of the STATA.

## RESULTS

Current smokers were 47.7% in males and 13.7% in females. The percentages according to age group are shown in Table 1. The genotype frequency for *DRD2 Taq*I A was in Hardy-Weinberg equilibrium for both sexes (*χ*^2^=0.861, p=0.353 for males and *χ*^2^=0.868, p=0.352 for females). When both sexes were combined, *A1A1* genotype was 12.9%, *A1A2* 46.2%, and *A2A2* 41.0%.

**Table 1.** Sex and age distributions of the subjects according to smoking status.

Age	Males				Females			
	Current	Former	Never	Total	Current	Former	Never	Total
20 - 29	7	1	7	15	6	3	22	31
(%)	(46.7)	( 6.7)	(46.7)	(100)	(19.4)	( 9.7)	(71.0)	(100)
30 - 39	10	5	7	22	13	5	51	69
(%)	(45.5)	(22.7)	(31.8)	(100)	(18.8)	( 7.3)	(73.9)	(100)
40 - 49	30	13	12	55	18	7	83	108
(%)	(54.6)	(23.6)	(21.8)	(100)	(16.7)	( 6.5)	(76.9)	(100)
50 - 59	60	23	20	103	18	9	102	129
(%)	(58.3)	(22.3)	(19.4)	(100)	(14.0)	( 7.0)	(79.1)	(100)
60 - 69	43	43	23	109	4	2	75	81
(%)	(39.5)	(39.5)	(21.1)	(100)	( 5.0)	( 2.5)	(92.6)	(100)
70 - 79	15	22	5	42	2	0	27	30
(%)	(35.7)	(52.4)	(11.9)	(100)	( 6.9)	( 0.0)	(93.1)	(100)

Total	165	107	74	346	61	26	360	447
(%)	(47.7)	(30.9)	(21.4)	(100)	(13.7)	( 5.8)	(80.5)	(100)

As shown in Table 2, the percentage for current smokers was lowest among males with *A1A1* genotype (35.3%), and highest among males with *A2A2* genotype (57.0%). However, the percentage was larger among females with *A1A1* than among females with *A2A2* genotype (19.6% and 10.9%, respectively). When the two groups were combined, the difference was cancelled.

**Table 2.** Smoking status according to sex and genotypes of *DRD2 Taq*I A and *Mbo*I polymorphisms.

	Smoking								Genotype %
	Current		Former		Never		Total		
*Taq*I A polymorphism									
Males									
*A1A1*	18	(35.3)	19	(37.3)	14	(27.5)	51	(100)	14.7
*A1A2*	66	(43.1)	55	(36.0)	32	(20.9)	153	(100)	44.2
*A2A2*	81	(57.0)	33	(23.2)	28	(19.7)	142	(100)	41.0
Females									
*A1A1*	10	(19.6)	3	( 5.9)	38	(74.5)	51	(100)	11.4
*A1A2*	31	(14.6)	13	( 6.1)	169	(79.3)	213	(100)	47.7
*A2A2*	20	(10.9)	10	( 5.5)	153	(83.6)	183	(100)	40.9

*Mbo*I polymorphism									
Males									
*AA*	60	(38.2)	63	(40.1)	34	(21.7)	157	(100)	90.8
*AT*	7	(43.8)	5	(31.3)	4	(25.0)	16	(100)	9.2
*TT*	0	( - )	0	( - )	0	( - )	0	( - )	0.0
Females									
*AA*	24	(12.4)	19	( 9.8)	150	(77.7)	193	(100)	87.3
*AT*	4	(15.4)	1	( 3.9)	21	(80.8)	26	(100)	11.8
*TT*	0	( 0.0)	0	( 0.0)	2	(100.0)	2	(100)	0.9

The ORs relative to *A1A1* genotype were calculated in four different settings of case-control design; current smoker cases compared with non-current (former + never) smoker controls, ever (current + former) smoker cases with never smoker controls, current smoker cases with never smoker controls, and current smoker cases with former smoker controls (Table 3). The age-adjusted OR of *A2A2* genotype was significantly elevated for current smokers compared with non-current or never smokers in males; OR=2.33 (1.19-4.53) with non-current smokers and OR=2.32 (1.02-5.29) with never smokers. The OR of current smoker cases with former smoker controls was also significant for *A2A2* genotype in males. The corresponding ORs for *A1A2* were almost in the mid of that for *A2A2*. In females, there were no significant ORs observed, though the ORs for *A2A2* genotype were less than unity and smaller than for *A1A2* genotype.

**Table 3.** Odds ratios (ORs) and 95% confidence intervals (95% CIs) of *DRD2 Taq*I A polymorphism

Genotype	Current vs. non-current				Ever vs. never				Current vs. never				Current vs. former			
	cOR^a^ (95% CI)		aOR^b^ (95% CI)		cOR^a^ (95% CI)		aOR^b^		cOR^a^ (95% CI)		aOR^b^ (95% CI)		cOR^a^ (95% CI)		aOR^b^	
Males																
*A1A1*	1	(Reference)	1	(Reference)	1	(Reference)	1	(Reference)	1	(Reference)	1	(Reference)	1	(Reference)	1	(Reference)
*A1A2*	1.39	(0.72-2.68)	1.37	(0.71-2.66)	1.43	(0.69-2.96)	1.47	(0.71-3.06)	1.60	(0.71-3.63)	1.61	(0.71-3.46)	1.27	(0.61-2.65)	1.19	(0.56-2.53)
*A2A2*	2.43	(1.25-4.73)	2.33	(1.19-4.53)	1.54	(0.73-3.23)	1.70	(0.79-3.56)	2.25	(0.99-5.11)	2.32	(1.02-5.29)	2.59	(1.21-5.55)	2.41	(1.11-5.26)

Females																
*A1A1*	1	(Reference)	1	(Reference)	1	(Reference)	1	(Reference)	1	(Reference)	1	(Reference)	1	(Reference)	1	(Reference)
*A1A2*	0.70	(0.32-1.54)	0.73	(0.33-1.62)	0.76	(0.37-1.55)	0.79	(0.39-1.64)	0.70	(0.31-1.54)	0.72	(0.32-1.61)	0.72	(0.17-3.03)	0.71	(0.16-3.11)
*A2A2*	0.50	(0.22-1.16)	0.52	(0.22-1.20)	0.57	(0.27-1.20)	0.59	(0.28-1.25)	0.50	(0.21-1.15)	0.51	(0.22-1.18)	0.60	(0.13-2.68)	0.60	(0.13-2.70)

^a^Crude odds ratio.

^b^Age-adjusted odds ratio.

CI: confidence interval.

Genotyping of *Mbo*I polymorphism was conducted for 395 participants including one individual whose DNA was not amplified by PCR-CTPP. As shown in Table 2, the great majority had the *AA* genotype. The distribution was in Hardy-Weinberg equation (*χ*^2^=0.407, p=0.524 for males, and *χ*^2^=1.090, p=0.297 for females). There was no association with smoking habits. Accordingly, no further analysis was conducted. Fifty-six individuals with *A1A1* genotype of *Taq*I A were all with *AA* genotype of *Mbo*I, while 128 (81.5%) out of 157 with *A2A2* had the *AA* genotype and 166 (91.7%) out of 181 with *A1A2* had the AA genotype.

## DISCUSSION

This is a confirmatory study for the hypothesis that smoking habits are associated with the *A2A2* genotype in Japanese. We estimated the sample size for the comparison between *A1A1* and *A2A2* as follows; two-sided alpha error = 0.05, statistical power 90%, from the previous study, smokers 12% (5/41) for *A1A1* and 29% (39/136) for *A2A2*, and genotype frequency, 0.123 for *A1A1*, 0.467 for *A1A2*, and 0.410 for *A2A2*, resulting in 692 participants (85, 323 and 284, respectively). Since the male/female ratio and smoking percentage were unknown, the sample size was set to be 800 at the start of the present study. The genotype frequency in this study (12.9% for *A1A1*, 46.2% for *A1A2* and 41.0% for *A2A2*) was similar to that for the previous study as mentioned above (n=332). The percentage of current smokers was slightly higher in males (47.7%) and same in females (13.7%) as the previous study (34.2%, n=155, and 13.6%, n=177, respectively). These figures indicated that the sample size estimation was appropriate.

The hypothesis that the polymorphism is associated with smoking habits was not confirmed when both sexes were combined. However, the findings for males were quite consistent. The association for females was not significant in the previous study. The OR was a little smaller than the previous study (OR=3.72 for current vs. non-current smokers), but this study with a larger sample size demonstrated a significant association with the *A2* allele. The p-value for OR=2.33 for current vs. non-current smokers was 0.013, so the adjustment of multiple comparisons for three tests (analysis for males, females, and combined) allows a significant result (0.013 × 3=0.039 < 0.05). Accordingly, it could be concluded that the association exists for males. In the present dataset, male current smokers also showed a significant OR compared with former smokers. Since this finding was not hypothesized in this study, further studies are required to evaluate the effect of the genotype on quitting smoking. There are several different reasons to quit smoking, the reasons should be taken into account in such studies.

Table 4 summarizes brief results from all the past studies on *DRD2 Taq*I A polymorphism with smoking habits. Since the ORs were not described on the papers, crude ORs were calculated from the figures in the table or text of each report. The first two reports by Noble et al.^2^ and Comings et al.^3^ showed a significantly reduced OR for *A2A2* genotype relative to *A1A2/A1A2* combined. The *Taq*I A genotype distribution reported by Spitz et al.^4^ was insignificantly associated with smoking habits, but the *Taq*I B linked to *Taq*I A was associated significantly. Singleton et al. reported that the *A2A2* allele was associated with current smoking,^12^ though not significant. Our two studies showed a similar, but significant finding for males that the *A2* allele was associated with smoking habits.

**Table 4.** Case-control studies on the association between smoking habits and *dopamine receptor D2 Taq*I A polymorphism

Authors (Country, year) Race	Subjects	Smoking Nev/for/cur ^a^	A2 allele (%)	Cases / controls	Age-adjusted OR (95% Confidence interval)		
					*A1A1*	*A1A2*	*A2A2*
Noble, et al. (US, 1994) Caucasians	All (n=354)	182/115/57	80.4	Current / never		1 ^b^	0.46 (0.24-0.90)
				Ever / never		1 ^b^	0.58 (0.31-1.08)
						(Crude OR calculated from the text)	

Comings, et al. (US, 1996) Caucasians	All (n=1,036)	714/312 ^c^	51.3 ^d^	Smokers / controls		1 ^b^	0.37 (0.28-0.49)
			74.1 ^d^			(Crude OR calculated from Table 1)	
		

Spitz, et al. (US, 1998) Caucasians	All (n=126)	13/67/46	ND ^c^	Ever / never		1 ^b^	0.50 (0.08-2.10)
						(Crude OR calculated from Table 2)	
	

Singleton, et al. (UK, 1998) Caucasians	All (n=221)	117/104 ^f^	79.9	Current / non-current		1 ^b^	1.68 (0.93-3.04)
						(Crude OR calculated from Table 1)	
	

Yoshida, et al. (Japan, 2001) Japanese	All (n=332)	198/57/77	64.3	Current / never	1	1.90 (0.63-5.70)	3.72 (1.23-11.2)
				Ever / never	1	1.65 (0.67-4.04)	3.68 (1.50-9.05)
	Males (n=155)	51/51/53	69.0	Ever / never	1	1.27 (0.43-3.77)	3.19 (1.06-9.60)
	Females (n=177)	147/6/24	60.2	Ever / never	1	3.58 (0.43-29.8)	7.59 (0.91-63.4)

Current study Japanese	All (n=793)	434/133/226	64.1	Current / never	1	1.10 (0.59-2.06)	1.09 (0.58-2.03)
				Ever / never	1	1.06 (0.62-1.83)	0.94 (0.54-1.63)
	Males (n=346)	74/107/165	63.2	Current / never	1	1.61 (0.71-3.46)	2.32 (1.02-5.29)
				Ever / never	1	1.47 (0.71-3.06)	1.70 (0.79-3.56)
	Females (n=447)	360/26/61	64.8	Current / never	1	0.72 (0.32-1.61)	0.51 (0.22-1.18)
				Ever / never	1	0.79 (0.39-1.64)	0.59 (0.28-1.25)

^a^Never/former/current smokers, ^b^*A1A1/A1A2*, ^c^Controls/smokers; smoking habit for the controls were not clearly described, ^d^51.3% for controls 74.1% for smokers, ^e^Not described, and ^f^Non-smokers/smokers. OR: odds ratio.

Background on biological mechanisms for the *DRD2* polymorphisms are scarce with smoking behavior, so the discussion based on biology may be very hypothetical.^1^ A plausible explanation is that the nicotinic receptors present on the dopaminergic cell bodies increase dopamine release in the nucleus accumbens of the mesolimbic system.^24^ It induces pleasurable feelings in smokers, and possibly the strength of pleasure depends on some genetic factors, which affects the smoking behavior. A significant reduction on DRD2 receptor availability (probably density) was observed in *A1A2* group compared with *A2A2* group.^25^ Bromocriptine, a DRD2 agonist, was effective to improve craving and anxiety only for alcoholics carrying *A1* allele.^26^ The associations with *DRD2* polymorphisms have been reported for many conditions including alcoholism,^26^^,^^27^ drug abuse,^28^^,^^29^ and obesity.^30^ These findings provide circumstantial evidence that the *DRD2* polymorphism also play a role in smoking behavior, though the direct mechanism relating to the behavior remains to be elucidated.

Social and/or psychological explanation for the inconsistent findings is also very hypothetical. Female smokers seem to be different from male smokers in terms of motivation to start smoking or to quit smoking at least in Japan. It may cause the difference in the ORs between males and females. Concerning the inconsistency among ethnic groups, genetic explanation should be also considered. The links to other functional polymorphisms or to polymorphisms of other genes may provide an answer to the inconsistent findings, though it does not explain the inconsistency between the studies of the United States and United Kingdom.

Among the 20 known polymorphisms of *DRD2*, *Taq*I A, *Taq*I B, and -141C Ins/Del have been examined concerning smoking habits.^2^^-^^4^^,^^12^^,^^15^ The link between *Taq*I A and *Taq*I B was reported to be stronger in Japanese^15^ than in Caucasians.^4^ The -141C Ins/Del was not related to smoking habits in Japanese.^15^ This study added a new finding that *Mbo*I was not related with smoking habits and a potential link with *Taq*I A polymorphism. There is no information on the links of *Taq*I A to the other genes.

In conclusion, the original hypothesis for both sex combined was not confirmed, however, the consistent results for males strongly indicated that the *A2* allele is associated with smoking habits for Japanese males. In females, the association was not observed in the present study, which suggested that smoking behavior might be affected differently in biological and/or psychological manner. The difference in the influence of *DRD2 Taq*I A polymorphism between males and females should further be examined for Japanese.
